# Sound check, stage design and screen plot – how to increase the comparability of fear conditioning and fear extinction experiments

**DOI:** 10.1007/s00213-018-5111-5

**Published:** 2018-11-23

**Authors:** Carsten T. Wotjak

**Affiliations:** Max Planck Institute of Psychiatry, RG “Neuronal Plasticity”, Kraepelinstr. 2-10, 80804 Munich, Germany

**Keywords:** Fear conditioning, Fear extinction, Reinstatement, Renewal, Spontaneous recovery, Return of fear

## Abstract

In the recent decade, fear conditioning has evolved as a standard procedure for testing cognitive abilities such as memory acquisition, consolidation, recall, reconsolidation, and extinction, preferentially in genetically modified mice. The reasons for the popularity of this powerful approach are its ease to perform, the short duration of training and testing, and its well-described neural basis. So why to bother about flaws in standardization of test procedures and analytical routines? Simplicity does not preclude the existence of fallacies. A short survey of the literature revealed an indifferent use of acoustic stimuli in terms of quality (i.e., white noise vs. sine wave), duration, and intensity. The same applies to the shock procedures. In the present article, I will provide evidence for the importance of qualitative and quantitative parameters of conditioned and unconditioned stimuli for the experimental outcome. Moreover, I will challenge frequently applied interpretations of short-term vs. long-term extinction and spontaneous recovery. On the basis of these concerns, I suggest a guideline for standardization of fear conditioning experiments in mice to improve the comparability of the experimental data.

## Introduction

The confrontation with threatening stimuli and situations bears an enormous risk for the survival of animals. Fundamental principles ensure that they may anticipate and avoid them. This includes defensive reflexes (e.g., fight, flight, freezing, startle reaction), which are known as fear responses. Moreover, they may cause learning processes which enable anticipatory responses for cases when a threatening situation is again encountered. Ivan P. Pavlov (1849–1936) (Rozo and Rodriguez-Moreno 2015) has developed a general theoretical framework for stimulus–stimulus associations (even though not in the context of fear) and coined the technical terms which are still in use today and at the basis of our understanding of those processes (Pavlov 1927).^1^ A PubMed search with the keywords “fear conditioning” revealed more than 7000 publications since 2000 with a steady increase until today. Not only acquisition but also extinction gained more and more attention, likely because of its potential translational value for anxiety therapy in human patients (Bowers and Ressler 2015; Lonsdorf et al. 2017; Singewald et al. 2015).

Recently, there was a number of excellent review articles published, which describe fear conditioning and fear extinction (Dejean et al. 2015; Ehrlich et al. 2009; Maren and Holmes 2016; Myers and Davis 2002, 2007; Pape and Pare 2010; Tovote et al. 2015) at anatomical and cellular levels. Not to forget about critical concerns of our current nomenclature and ongoing controversies about what to name fear and what not (Fanselow and Pennington 2018; LeDoux 2014, 2017). It is far beyond the scope of this article to enter such fundamental discussions. Rather, I will focus on procedural aspects and further narrow this down to studies in mice to come up with a guideline for standard fear conditioning and fear extinction experiments which are aimed at screening for behavioral phenotypes caused by pharmacological and/or genetic interventions. The importance of standardized stage design and screen plots for the interpretability of the experiments goes back to the work of Pavlov and his fellows (Podkopajew 1956), but receives surprisingly little attention today. The recommendations are certainly biased by own experiences and only scratch the surface of the theoretical framework which underlies Pavlovian conditioning. They are meant as suggestions for experimental paradigms in the exploration phase of projects on stress-, drug-, or gene-related changes in fear memory acquisition, consolidation, retention, and extinction. For sure, carryover effects of prior tests are likely to emerge. However, the test batteries described will help to reduce the number of experimental subjects while refining the interpretation of the data and enhancing the comparability between different labs.

## Cued vs. contextual conditioning

**Fig. 1:**
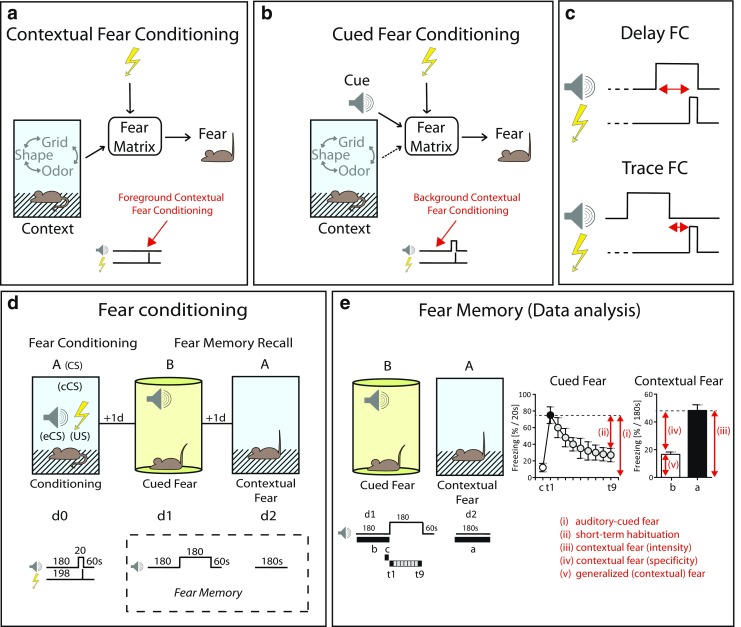
Principles of fear conditioning. Fear conditioning (FC) can be based on protocols without (**a**) and with explicit pairing (**b**) of a discrete sensory stimulus with a foot shock, whereby stimulus (CS) and electric shock (US) may overlap or be separated by a temporal gap (**c**). In a prototypic FC experiment (**d**), mice receive a single tone–shock pairing in the conditioning context (A), followed by re-exposure to the tone in a different test context (B) followed by re-exposure to the original conditioning context (A), each separated by 24 h. Analysis of conditioned fear (**e**) has to consider that freezing to the tone should be analyzed in time intervals (*t1–t9*) corresponding to the duration of the tone during conditioning and include freezing during baseline (i.e., during the last time interval just preceding tone onset, *c*). (i) Direct comparison of *t1* with *c* serves as a measure of tone-associated fear. (ii) In case of prolonged tone presentations, the development of the freezing response from *t1* to *t9* indicates short-term fear relief (i.e., habituation). (iii) The intensity of contextual fear is indicated by the freezing response in the conditioning context averaged of the entire exposure (i.e., 3 min: *a*). (iv) The specificity of contextual fear is deduced from direct comparison of freezing in the conditioning context (*a*) vs. baseline freezing in the test context (*b*). (v) Baseline freezing should be normalized to the same observation interval as freezing in the conditioning context (e.g., 3 min) and serves as a measure of generalized fear

A given aversive encounter may result in multiple associations of elemental or conjunctive representation of multiple configural cues with a threat. Elemental stands for discrete sensory stimuli of visual, olfactory, tactile, or acoustic nature. Configural, in contrast, describes the association of temporally and spatially discrete elemental stimuli (possibly with information about internal state of the animals) into a conjunctive holistic representation of the test situation, called context (Fanselow 2010; Maren et al. 2013; Rudy et al. 2004; Wallenstein et al. 1998) (Fig. 1a, b).

The primary difference between fear conditionings with elemental vs. configural cues lays in the role of the hippocampus formation: whereas elemental conditioning can go without it, configural conditioning essentially depends on it (Fanselow 2010; Maren et al. 2013). There is evidence that the role of the hippocampus is to “binding together” the elemental cues, i.e., to form a contextual representation of the test situation. This does not necessarily include local association with the aversive stimulus, which seems to happen at level of the basolateral amygdala (Fanselow 2010; Kim and Fanselow 1992; Yaniv et al. 2004). Threat and aversive encounters only indirectly influence contextual fear conditioning at level of the hippocampus via stress hormones which may affect synaptic plasticity related to context representation (Hagena et al. 2016; Joels et al. 2011; Sandi 2011). A reduced activation of the hormonal stress axes due to prior handling or surgical interventions might be responsible for the lower levels of conditioning observed after such interventions.

Cued and configural conditioning seem to represent complementary principles, which, sometimes, show antagonistic features. The latter applies not only to recall but also to encoding of the fear memory (Desmedt et al. 1999; Rudy et al. 2004). In a given situation, mice may associate an explicit cue, the context or both with an electric shock. The preponderance of the different memory traces depends on the conditioning protocol and the passage of time after conditioning (Sacco and Sacchetti 2010). The context may stand in the “background” to an explicit cue—shock association (Fig. 1b) or not (“foreground”). In the latter case, enhanced freezing upon re-exposure to the conditioning context not necessarily indicates contextual memory (Gerlai 1998), since mice may reduce the complex test situation to its elemental components with the consequence that elemental cues (e.g., grid floor, odor) will form an association with the shock, thus mimicking contextual fear memory (Golub et al. 2009; Rudy et al. 2004).

Many experiments are performed with limited availability of mutant mice. Moreover, ethical concerns require a reduction of animal numbers while refining experimental procedures. To keep with these requirements, it would be optimal to measure both elemental and configural association within the same animals in one experimental series. This can be achieved with a single pairing of an explicit cue with an electric shock with subsequent exposure to the cue in a neutral environment following exposure to the original conditioning chamber to measure contextual fear memory (Fig. 1d). However, there might be considerable line differences in the formation of background contextual fear memory. Anyhow, one has to keep in mind that the salience of the background context decreases with the number of associations between the explicit cue and the electric shock.

By definition, conditioning procedures with presentation of cue and shock without a time gap in between are termed *delay fear conditioning* (Fig. 1c). In contrast, in *trace fear conditioning*, there is a time gap between end of tone and onset of the shock (typically between 5 and 30 s; Fig. 1c). The latter procedure requires multiple tone-shock presentations seems to crucially depend on the hippocampus formation and the prefrontal cortex (Buchel et al. 1999; Gao et al. 2010; Gilmartin et al. 2014; Guimarais et al. 2011; Wanisch et al. 2005). In case of multiple stimulus–shock pairings, contingency comes into play which describes the ability (i.e., probability) for the stimulus to predict the occurrence of the electric shock. In basic research, 100% reinforcement is standard (i.e., the foot shock follows the stimulus in each case).

## Elemental stimuli

In line with the definition of Pavlovian conditioning, a priori neutral sensory stimuli acquire the ability to predict an adverse consequence upon association with an electric shock. In principle, stimuli of all sensory modalities might be employed, i.e., visual, acoustic, olfactory, and tactile stimuli. On closer inspection of the literature, it becomes clear that auditory stimuli predominate by far. Why is this the case?

Upon the first glance, *olfactory stimuli* have the highest ecological relevance for macrosmat animals like mice (Otto et al. 2000). However, in common laboratory settings, it is very challenging to restrict olfactory cues to the conditioning and/or test context without contaminating the room air, and to ensure proper temporal relationships between olfactory cue and electric shock. Thus, olfactory stimuli are mostly employed as a distinct component of the conditioning context and as that associated with the foot shock (Pamplona et al. 2011). However, if one can cope with the technical challenges, odors are highly suitable as elemental cues and provide important insights into memory processes including their transgenerational “inheritance” (Dias and Ressler 2014). Importantly, olfactory stimuli should be a priori neutral to the animals, irrespective of whether included into the background context or used as an explicit cue. According to a recent study (Root et al. 2014), isoamyl acetate would be a perfect candidate. According to our own experience, the commonly used ethanol (≥ 70%) bears the risk of being aversive to the animals from the beginning (Pamplona et al. 2011).

Also, *visual stimuli* turned out to be fairly unsuitable for conditioning experiments in mice, at least if evidence for the formation of fear memory solely relies on freezing behavior. This might be ascribed to evolutionary constraints due to the preferentially nocturnal activity of the animals. Other than for acoustic stimuli, a direct thalamic relay to the amygdala is missing for visual stimuli. It is tempting to assume that without such connection, visual stimuli hardly provoke prototypic fear responses, such as freezing and—in striking contrast to rats—potentiation of the startle response after a few pairing with the foot shock. A remarkable study supports this scenario: neonatal lesions of the inferior colliculus in mice caused rewiring of visual projections to the auditory thalamus. In consequence, mice could be readily conditioned with a few light cue–shock pairings to show enhanced freezing upon re-exposure to the visual stimulus (Newton et al. 2004).

For *acoustic stimuli*, in contrast, there are both cortical and thalamic inputs to the amygdala (Medina et al. 2002). This might explain why acoustic stimuli seem to be “prepared” for causing a freezing response even in naïve animals, presupposed they exceed a certain loudness (Kamprath and Wotjak 2004). As a consequence, acoustic stimuli cannot be regarded as being a priori “neutral,” but rather subliminal in terms of eliciting fear responses. This sounds like a pure academic concern but helps to understand that LTP-like processes may enhance their fear-provoking consequences in preexisting pathways (Nabavi et al. 2014; Rogan and LeDoux 1995; Rogan et al. 1997).

### Sound check: critical considerations about auditory fear conditioning

The comparison of the different sensory modalities used as CS in fear conditioning paradigms has revealed acoustic stimuli to appear best suited for standard experiments in mice. Very simplistically, acoustic stimuli may include simple sine wave tones with fixed frequency and amplitude, complex tones with altered frequency and amplitude, complex sounds composed by sine wave patterns, and white/pink noise. It is far beyond the scope of this article to go into details of psychoacoustics and its neuronal correlates. I rather will focus on the most frequently used acoustic stimuli (pure sine wave tones with fixed frequency/amplitude and white noise) and discuss their pros and cons for fear conditioning experiments.

**Fig. 2:**
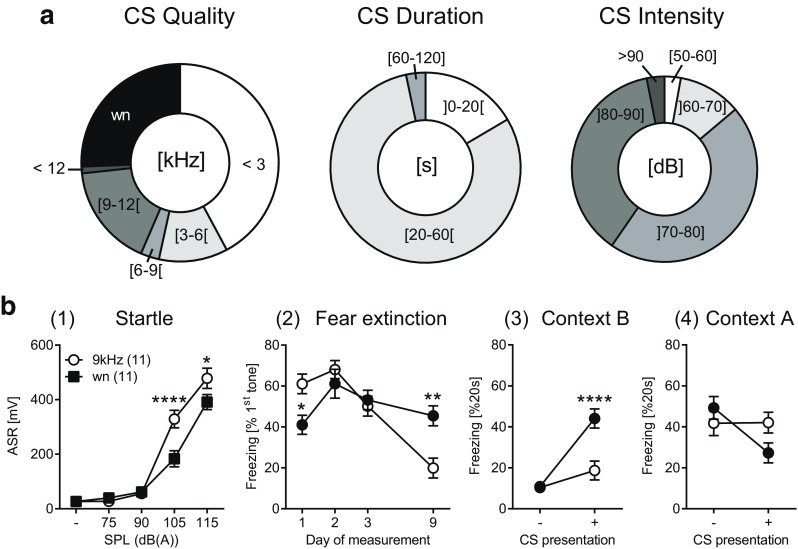
Variability in parametric settings of auditory stimuli affect fear conditioning experiments. **a** A literature survey on fear conditioning experiments in mice (> 350 studies performed between 2000 and 2010) revealed a broad variability in quality (frequency of sine-wave tones vs. white noise (wn)), duration and intensity of the conditioned stimuli (CS). **b** Direct comparison of consequences of white noise (wn) vs. sine wave (sw 9 kHz, 80 dB) stimuli on auditory fear conditioning and fear extinction. (1) Before conditioning, we compared the sensitivity of single-house male C57BL/6N mice (*n* = 11 per group) to startle pulses of different quality (white noise vs. sine wave) and intensity and observed in increased sensitivity to the sine wave tone as measured by acoustic startle responses (ASR; two-way rmANOVA, CS quality × SPL: *F*_4,80_ = 7.163, *p* < 0.0001). On basis of these data we selected an intensity of 80 dB for the subsequent fear conditioning with a single CS (20 s) -shock (0.7 mA, 2 s) pairing using the delay protocol (cf. Fig. 1c) in context A. (2) The day after fear conditioning, we placed the animals into a novel context (B) and repeatedly exposed them to nine CS (20 s, variable interval 30–140 s) per day on three consecutive days (d1–d3). We assessed retention of fear extinction by presenting another 4 CS to the animals at day 9. Long-term extinction was analyzed on basis of the freezing responses shown during the first tone presentation per day (cf. Fig. 3b; CS quality × day: *F*_3,60_ = 11.17, *p* < 0.0001), indicating the success of extinction training with sine wave but not with white noise stimuli. (3) This conclusion was substantiated, if freezing just before the first tone presentation (CS−) at day 9 was compared with freezing during the  subsequent 20-s tone presentation (CS+), whereby the white noise, but not the sine wave stimulus still caused a sharp increase in freezing (CS quality × CS: *F*_1,22_ = 30.69, *p* < 0.0001). (4) The situation was different, if animals were exposed to the CS in the conditioning context. Both groups showed the same level of contextual fear before CS presentation. This time, however, presentation of the white noise caused a decrease in freezing compared to the sine wave stimulus (CS quality × CS: *F*_1,22_ = 4.968, *p* = 0.0364), likely because of an explosive outburst of active fear (Fadok et al. 2017)

A survey of the literature between 2000 and 2010 revealed a broad range of tone frequencies and intensities in studies on auditory fear conditioning in mice (Fig. 2a). In particular, the large number of studies employing tone frequencies ≤ 6 kHz is remarkable, given the hearing capabilities of mice: as confirmed in multiple studies with different physiological read-outs, including inner ear measurements and measurements of brain stem responses, mice are most sensitive for tone frequencies between 10 and 20 kHz (Heffner and Heffner 2007; Marsch et al. 2007). At lower frequencies, tone intensity has to be substantial in order to evoke a comparable intracerebral response. For instance, in a differential fear conditioning paradigm which involved sine wave tones of 1 kHz vs. 7.5 kHz, we needed 95 dB vs. 85 dB in order to evoke a similar 50% maximal response of auditory-evoked potentials in C57BL/6JOlaHsd mice (Tang et al. 2003). As shown in Fig. 2a, a remarkably high number of studies employed tone frequencies and intensities which should have caused significant problems in perception, but apparently have not. To resolve this conundrum, we have to envision that tone intensities are not always validated by explicit measurements, and sine wave tones of low frequencies may include harmonics of higher frequencies which fall within the best hearing range of the animals. In particular, in experiments employing different tones (i.e., differential fear conditioning), tone frequencies have to be in a comparable sensitivity range in order to permit comparability of the outcome. Intuitively, one would go for frequencies > 10 kHz. This, however, precludes proper perception by the experimenter, since such frequencies may easily exceed human hearing capabilities. Moreover, also recording of the conditioned stimuli would be limited by physical characteristics of conventional cameras and microphones, as it is the case for tone presentations with common loud speakers. Therefore, standard experiments should use frequencies approaching the lower boundary of the best frequency range of mice (i.e., 9 to 10 kHz). Even under those conditions, scientists have to consider line differences in tone perception (Stiedl et al. 1999) and the emergence of hearing loss during aging (Ison et al. 2007).

It is also of note that tone intensities (and quality) may vary considerably depending on the quality of the speakers, their placement in the setup, and characteristics of the behavioral apparatus: First, one has to consider the inertia of the membrane which may result in artificial acoustic stimuli upon on-set and off-set of the tones. Such artifacts might be reduced by including rising and falling times (10–20 ms) in the electrical pulses to the speakers instead of using simple rectangular pulses. Second, tone intensities may vary considerably, depending on the installation of speakers relative to the setup and its dimensions. The size of the conditioning/test environment plays an important role for the display of different defensive responses. This may relate—in part—to differences in tone intensities due to differences in the distance to the speakers. Preferentially, the speaker is mounted centrally above the arena to reduce the incidence of intensity gradients. This has to apply to both conditioning and test contexts. Third, it is practically impossible to avoid sound reflections in any context. One has to be aware of this fact if different floors (grid floor, plain floor, or coverage of the floor with bedding/sand) are used. One way to minimize such differences could be to equalize the floor conditions as far as possible for instance by introducing bedding not only into the test setup but also into the waste bin of the conditioning setups beneath the grid floor.

White noise represents an alternative to sine wave tones. Many commercially available fear conditioning systems allow to choose between sine wave tones and white noise. This presupposes that all acoustic stimuli are equal/comparable if it comes to their application to fear conditioning experiments, which, however, is not the case. We designed a small set of experiments (Fig. 2b) in which we compared startle responses, conditioned fear, and fear extinction elicited by a sine wave tone (9 kHz, 80 dB) vs. white noise (80 dB). Both, sine wave tone and white noise revealed a similar relationship between stimulus intensities and startle responses with a slightly higher sensitivity to the sine wave tone. Mice also showed a higher freezing response to the sine wave tone than to the white noise burst, 1 day after a single pairing of the acoustic stimulus with an electric foot shock (0.7 mA, 2 s). Extinction training by repeated exposures to the stimuli, however, failed to cause any decline for white noise, which was in striking contrast to re-exposure to the sine wave tone (Fig. 2b2/3). We can conclude from these data that there are considerable differences in conditioned fear evoked by sine wave tones vs. white nose which require particular attention and ask for standardization of the experiments. I wish to argue for the use of sine wave tones, which offer the possibility for discriminatory fear conditioning paradigms with multiple conditioned stimuli (paired, neutral, or unpaired with the foot shock) which differ in tone frequency.

## Unconditioned stimulus

Most experiments use electric shocks as unconditioned stimuli, and only very few employ other (e.g., air puffs). In the recent years, our knowledge about brain circuits involved in perception of the shock has been substantially broadened (Johansen et al. 2010; Kim et al. 2013). It is self-explaining that conditioned fear depends on the shock intensity. This becomes evident among others from the composition of defensive responses evoked upon recall of the fear memory (i.e., risk assessment vs. freezing vs. flight reactions) (Laxmi et al. 2003). In addition, conditioned fear seems to involve not only associative components (which directly result from stimulus–shock associations) but also nonassociative components (which merely represent a general increase in evoked fear due enhanced responsiveness of the fear circuit after perception of the shock, a phenomenon called fear sensitization; Kamprath and Wotjak 2004; Siegmund and Wotjak 2006, 2007). In some mouse strains (e.g., C57BL/6N), an increase in shock intensity seems to primarily affect fear sensitization (Kamprath and Wotjak 2004). This, however, was not the case in others (e.g., BALB/c) (Thoeringer et al. 2010). Furthermore, an unequivocal distinction between associative and nonassociative fear requires the assessment of low levels of generalized contextual fear, which has to be quantified for the time preceding tone presentation in the new test environment (Fig. 1e).

The choice of an appropriate shock intensity depends on numerous factors, including scientific rationale (e.g., physiological vs. pathological fear responses), mouse strain, and equipment. In particular, the latter hinders a direct comparison of different experiments. Upon first glance, this may astonish, since all shockers allow a clear setting of the current intensity. However, often it remains unknown to the scientist whether this setting considers potential differences in the resistance of the animals and applies to AC vs. DC, scrambled vs. non-scrambled shocks. A potential way out of this “mess” would be—without getting lost in physics—to express the shock intensities relative to the individual pain threshold of the animals under study. Pain thresholds should be assessed in the same experimental setting with experimentally naïve animals. There are two different ways to present the shocks, either as brief discrete rectangular pulses of increasing intensity or as a ramp of continuously increasing intensity. Both methods have their pros and cons: Discrete stimuli are more prone to evoke startle responses which might be mistaken for nociception. Ramps bear the risk of adaptation. Anyhow, readouts should distinguish between discomfort (i.e., backward moving) and pain responses (paw flicking vs. jumping/vocalization), and the shock intensities which elicit their first emergence will be noted (Siegmund et al. 2005; Thoeringer et al. 2010). One has to keep in mind that nociception is prone to phenomena such as stress-induced analgesia (Butler and Finn 2009; Lau and Vaughan 2014). Therefore, mice have to accommodate to the shock environment (e.g., ≥ 3 min, as done in standard conditioning procedures) before starting with the assessment of pain thresholds. Furthermore, one has to consider that, in particular, mutant mice may show deficits in expression of discomfort/pain responses. Therefore, one has to define maximal shock intensities in order to avoid physical harm to the animals.

Following these advices and given the remarkable variance in individual pain thresholds even among inbred mice, we can chose the shock intensity applied in subsequent fear conditioning experiments as multitude of the averaged pain threshold (Thoeringer et al. 2010). This would enable the direct comparison of experimental data obtained in different labs with different equipment. It is important to note the qualitatively different fear responses caused by shock intensities two times vs. three times the individual pain threshold in mutant (Kamprath et al. 2009) and inbred mice (Thoeringer et al. 2010). At the same time, conditioned fear nonmonotonically relates to the intensity of the foot shock (e.g., Davis and Astrachan 1978), thus demonstrating the complexity of the issue. Therefore, it is highly recommended (i) to report the pain thresholds measured in the same equipment as used for fear conditioning in addition to the shock intensity used for conditioning (to enhance the comparability of the data between different labs) and (ii) to consider different shock intensities with different cohorts of mice also in case of predefined pain thresholds. Prior aversive experiences (e.g., surgery) may change the impact of the electric shock. Therefore, shock intensities have to be adjusted accordingly, before starting with a laborious and time consuming experiment.

## Fear memory

Fear memories show multiple faces. In humans, we may distinguish between explicit and implicit memory components. Explicit fear memories encode the factual knowledge about the “danger” of a stimulus/situation. Implicit fear memories, in contrast, describe changes at level of the fear circuit^2^ which elicit an automatic reflexive fear response upon encounter of a potentially threatening stimulus/situation. As revealed in patients with selective brain lesions, both memory components are dissociable (Bechara et al. 1995; Feinstein et al. 2011). It is evident that we cannot make a similar distinction in mice. This does not preclude the coexistence of multiple memory systems also in animals. Rather, we lack adequate readouts and paradigms which would allow us to assess factual knowledge about the danger of a stimulus/situation. Therefore, the knowledge about conditioned fear in basic science solely relates to implicit fear memories.

Implicit fear memories are typically assessed on basis of fear responses evoked upon re-exposure to the conditioned stimuli. Fear responses could be of vegetative (i.e., relating to changes in autonomous nervous system such as heart rate, blood pressure of breathing frequency), hormonal (i.e., changes in stress hormone levels), or behavioral nature. The latter include risk assessment (Blanchard et al. 2011), freezing (Fanselow 1994), defensive burying (De Boer and Koolhaas 2003), fear potentiated startle (Walker et al. 2009), and ultrasonic vocalization (USV) (Kim et al. 2010). Not all of them are equally suited for experiments in mice (e.g., USV). As discussed before, aversive encounter may lead to associative and nonassociative changes in the fear circuit. Thus, quantitative differences in fear responses between two cohorts of mice have to be interpreted with caution. This applies in particular to studies which interpret differences in freezing as direct measure for differences in associative memory processes.

### Contextual fear memories

To measure contextual fear, mice are placed back to the original conditioning context, however without explicit cues or additional electric shocks (Fig. 1a). The duration of the exposure varies considerably between different labs. Preferentially, it should resemble at least the time until shock presentation during conditioning, keeping in mind that temporal aspects of the shock presentation might be part of the contextual memory. More and more scientists start to acknowledge that a strong fear response in the conditioning context is not necessarily maladaptive. Therefore, it is worthwhile to include additional context exposures with different test environments to assess not only intensity but also specificity of contextual fear (Balogh et al. 2002; Balogh and Wehner 2003; Golub et al. 2009; Maren et al. 2013) (Fig. 1e). Such additional encounters may include dominant features of the original shock context (e.g., olfactory cues, grid floor) or be totally distinct. The latter is barely achievable, given that the majority of the studies will be performed by the same experimenter in the same room and even inside the same isolation cubicles. This means, we should not neglect the potential effects of contextual reminders more remote from the test setup and relating, for instance, to the transportation procedure (Rudy et al. 2004). Moreover, if kept under standard housing conditions, mice are not used to grid floors or plain floors. The resulting level of discomfort (potentiated by nonassociative components of the fear memory) is reflected by a certain level of “baseline fear” even in the best designed “novel context” (Kamprath and Wotjak 2004).

If there are multiple test sessions in different environments (e.g., contextual vs. cued fear memory, expression, extinction, and renewal of conditioned fear), can this be done within the same animals without risking carryover effects which significantly affect the interpretability of the data? The answer is, it cannot. For instance, the order of context exposures matters a lot if it comes to the increase in context generalization with the passage of time (Wiltgen and Silva 2007). In any case, prior exposures very likely influence the behavioral outcome of the next. Consequently, test batteries cannot be interpreted in a straightforward manner and experiments should include multiple independent experimental groups. Preferentially, key findings have to be replicated in new cohorts of experimentally naive mice with only that key finding in focus. However, the necessary number of animals is rarely available (and sometimes at odds with the 3R rule of animal experimentation). Therefore, a standardized series of experiments within the same subjects would provide a first screening approach which not only keeps the number of animals at a minimum but also ensures comparability of data obtained in different labs. On the basis of own experiences and the literature, I recommend to start with the context most different from the original conditioning context, which is typically the test context for measuring fear responses to the elemental cue (Fig. 1d). The time before cue presentation may serve as a measure for generalized contextual fear (Fig. 1e). One has just to make sure that the period before cue presentation is similar to the duration of the subsequent re-exposure to the conditioning context. There should be some recovery time in between the two exposures (e.g., 24 h; Fig. 1d). This order of testing has an increased bias for generalized contextual fear (which is tested first). On the other hand, a more pronounced freezing response to the shock context than to the novel test context allows conclusions about both, specificity and intensity of contextual fear. In case of an opposite order, reduced freezing in the test context might partially reflect extinction of the fear memory caused by the prior re-exposure to the shock context rather than true context discrimination.

### Cued fear memories and conditioned responses

As mentioned before, the majority of studies measure auditory-cued fear memories in mice by assessing freezing behavior. For mice—other than for rats (Walker et al. 2009)—valid protocols for measurements of fear-potentiated startle responses with auditory (or visual) conditioned stimuli still remain to be established. This is a significant disadvantage, since measurements of fear-potentiated startle responses allow (i) bidirectional modulation of the fear response (e.g., conditioned stimuli may potentiate, whereas safety or relief learning may attenuate the startle response) (Gerber et al. 2014; Mayer et al. 2018), and (ii) dissection of “nonassociative” (relating to the intensity of the foot shock which may cause a general increase in arousal) and “associative memory” components (relating to the association between conditioned stimulus and foot shock) by comparing the startle response elicited by the test pulse with and without preceding presentation of the conditioned stimulus. There are recent attempts which successfully combined startle and freezing measures in order to distinguish between phasic vs. sustained fear responses in mice (Daldrup et al. 2015; Seidenbecher et al. 2016) thus closely resembling the scenario suggested for rats (Davis et al. 2010).

It is worthwhile to recollect that mice may show multiple different fear responses, depending on the shock intensity (Laxmi et al. 2003), the cue intensity (Kamprath and Wotjak 2004), and imminence of a threatening situation (McNaughton and Corr 2004; Perusini and Fanselow 2015). Also, chemical, electrical, or optogenetic stimulation in the same sensory afferences to the fear circuit may cause pre-encounter-like (e.g., risk assessment), post-encounter-like (e.g., freezing), and circa-strike-like responses (e.g., flight behavior) (Evans et al. 2018; Shang et al. 2015; Tovote et al. 2016) as introduced by Fanselow and Lester (1988). The possibility of such a switch between passive and active fear responses is largely neglected in fear conditioning experiments, with only a few exceptions (Fadok et al. 2017; Metna-Laurent et al. 2012). In concordance with the defensive distance concept (Brandao et al. 1994; McNaughton and Corr 2004; Perusini and Fanselow 2015), animals display risk assessment behavior to more distant but freezing or escape to more proximal cues. Larger arenas give the animals an option to withdraw from the aversive stimulus (as experienced by a decrease in stimulus intensity). Therefore, larger arenas favor the occurrence of risk assessment, whereas smaller arenas increase the preponderance of freezing behavior.

The assessment of freezing is rather trivial, since it is mostly synonymous with immobility. This has to be ascribed to difficulties in identifying crouching postures known from rats and to the fact that “freezing” is mostly automatically assessed by video tracking systems (Anagnostaras et al. 2000; Meuth et al. 2013) or infrared beams. For such cases, I strongly recommend to additionally record videos which also allow for subsequent offline analyses of other defensive responses, such as burying (De Boer and Koolhaas 2003), rearing (Lever et al. 2006), or risk assessment (Blanchard et al. 2011). “Immobility” measures might become corrupted by resting or sleeping, in particular during prolonged tone exposures (Cain et al. 2003). To reduce this risk, experiments might be performed (i) during the activity phase of the animals, and/or (ii) with bedding covering the floor of the test context (which stimulates active behaviors). It might also be of advantage to study the expression of conditioned fear in an operant task, where food- or water-deprived animals get access to food/water. This brings them into an active state, which is very sensitive to suppressive effects of CS presentations. Anyhow, the fact that fear comes in different flavors asks for a careful interpretation of the findings. By no means, the absence of freezing necessarily indicates the absence of fear! Studies on fear conditioning should acknowledge this caveat, e.g., by describing changes in the behavioral measure (“decrease in freezing”) rather than our interpretation of the observations (“decrease in fear”). Of course, most of these caveats also apply to the interpretation of contextual fear.

### A control is not a control is not a control

What makes fear conditioning so attractive is its simplicity to perform, its translational value and, not to forget, the fact that memories can be formed in a single learning trial. The latter enables the study of consolidation processes at defined time points after the learning event. Historically, there is a distinction between short-term and long-term fear memory, which is based on the independence vs. dependence on protein biosynthesis (Kandel 2001; McGaugh 2000). However, aside to the various experimental parameters outlined before, also the choice of appropriate controls represents a major constraint to the interpretation of the data. This applies, in particular, to the associative nature of the fear memory. Here is not the place for a comprehensive discussion of possible controls, which clearly depend on each specific experimental design and the scientific question. I would like to mention only a few critical issues which need to be considered while designing appropriate controls: for instance, many studies have involved explicit unpairing of tone and shock, assuming that this precludes associative learning processes. However, there is evidence that mice may instead form a tone–no shock association (i.e., “safety learning”) (Gerber et al. 2014; Kong et al. 2014; Pollak et al. 2010, 2008). This is reflected by auditory-evoked potentials measured in the lateral amygdala, where tone–shock pairing leads to long-lasting potentiation (Rogan et al. 1997; Tang et al. 2001, 2003), unpairing to long-lasting depression (Rogan et al. 2005; Tang et al. 2001), whereas auditory-evoked potentials remained at pre-conditioning levels in case of neutral tones (Rogan et al. 2005; Tang et al. 2003). These processes are particularly sensitive to repeated tone and shock presentations. In addition, explicit unpairing increases the salience of the “background” context, which now better predicts the occurrence of the electric shock, thus leading to confounding influences of contextual fear conditioning. This risk might be reduced by a phenomenon known as “immediate-shock deficit.” Hereby, administration of the foot shock right after insertion of the animals into the conditioning context prevents the formation of contextual fear memory, since the animals had not enough time to form an internal representation of the environment (Landeira-Fernandez 1996; Landeira-Fernandez et al. 2006). But even under those circumstances, animals may use the transport to the chamber to contextualize the conditioning procedure (Rudy et al. 2004). Moreover, the shock might be differentially perceived due to stress-induced analgesia (Butler and Finn 2009; Lau and Vaughan 2014) resulting from handling and novelty exposure.

### Data analysis—fear conditioning experiments

Also data analysis requires careful considerations. First, direct comparison of different freezing responses (e.g., shock context before conditioning vs. novel test context vs. re-exposure to the shock context) should be based on similar observation periods (Fig. 1e). It is not acceptable to express data as a percentage of total observation times to get rid of differences in the length of the analysis interval, since fear responses show distinct temporal dynamics over the course of the exposure. Second, such temporal dynamics are often very informative, in particular during expression of cued fear memories. To assess them, data have to be analyzed in rather short time intervals (Fig. 1e). Here, everything is possible but not necessarily meaningful. As a rule of thumb, I suggest that analysis intervals correspond to the duration of the conditioned stimulus during conditioning (mostly 10–30 s). Conditioned mice often show a mobility burst upon onset of auditory stimulus (Fadok et al. 2017) before they acquire an immobile posture. This could result in “false-negative fear memory,” if too short analysis intervals (< 10s) are chosen. In turn, longer durations of the CS (> 120 s) not only bear the risk of habituation during conditioning but also unnecessarily prolong the duration of extinction training sessions, which may result in resting or sleeping animals.

## As fear goes by

Fear conditioning experiments are employed to study not only cellular and molecular correlates of fear memory formation but also its disappearance. Again, it is far beyond the scope of this article to repeat the many comprehensive reviews on extinction (Ehrlich et al. 2009; Lonsdorf et al. 2017; Myers and Davis 2002; Riebe et al. 2012; Singewald et al. 2015; Tovote et al. 2016) or reconsolidation (Alberini and Ledoux 2013; Almeida-Correa and Amaral 2014; Bonin and De Koninck 2015; Clem and Schiller 2016; Nader and Hardt 2009; Riccio et al. 2006; Singewald et al. 2015). Yet, I wish to emphasize the existence of multiple (not necessarily mutually exclusive) processes, which may ultimately result in a decrease in conditioned fear, such as forgetting, disturbed systems consolidation, habituation, desensitization, deconsolidation, and extinction/relearning (Riebe et al. 2012; Singewald et al. 2015). Knowledge about them guides proper study design and, thus, improves the interpretation of the results. To classify them, we have to distinguish between processes which go without or with recall of the fear memory. Examples for the first would be *forgetting/disturbed long-term consolidation* or *erasure* of memory traces. For instance, fear memory undergoes systems consolidation, i.e., changes in anatomical and molecular underpinnings over the course of weeks after conditioning (Frankland and Bontempi 2005; Sacco and Sacchetti 2010; Tayler et al. 2013; Thoeringer et al. 2012; Vetere et al. 2017; Wheeler et al. 2013). Thus, pharmacological interventions during this period may affect long-term maintenance of the fear memory without re-exposure to the conditioning context (Thoeringer et al. 2012).

Recall of the fear memory can have multiple consequences. For instance, it may render already consolidated memories labile, so that they may undergo changes and/or get updated (for references: see above). This process was termed *reconsolidation* (another hotly debated term). Timed pharmacological interventions or sophisticated behavioral procedures may block this process and, thus, lead to deconsolidation and decay of conditioned fear. In any case, human studies suggest that deconsolidation primarily affects implicit components of the fear memory, leaving the explicit narrative component unaffected (Kindt et al. 2009). This renders such experiments perfectly suitable for studies in animals.

Repeated and/or prolonged recall of the fear memory in absence of the predicted shock leads to a relearning process called *fear extinction*. As a consequence mice form a new memory which suppresses the expression of the original fear memory. Importantly, fear extinction is context dependent (Bouton 2004; Bouton et al. 2006; Maren et al. 2013). Fear extinction training may trigger other processes such as *desensitization* or *habituation*, which also result in a decrease in conditioned fear, however, primarily by affecting nonassociative components of the fear memory (Kamprath et al. 2006; Kamprath and Wotjak 2004; McDiarmid et al. 2017; McSweeney and Murphy 2009; McSweeney and Swindell 2002; Poon and Young 2006; Riebe et al. 2012).

**Fig. 3:**
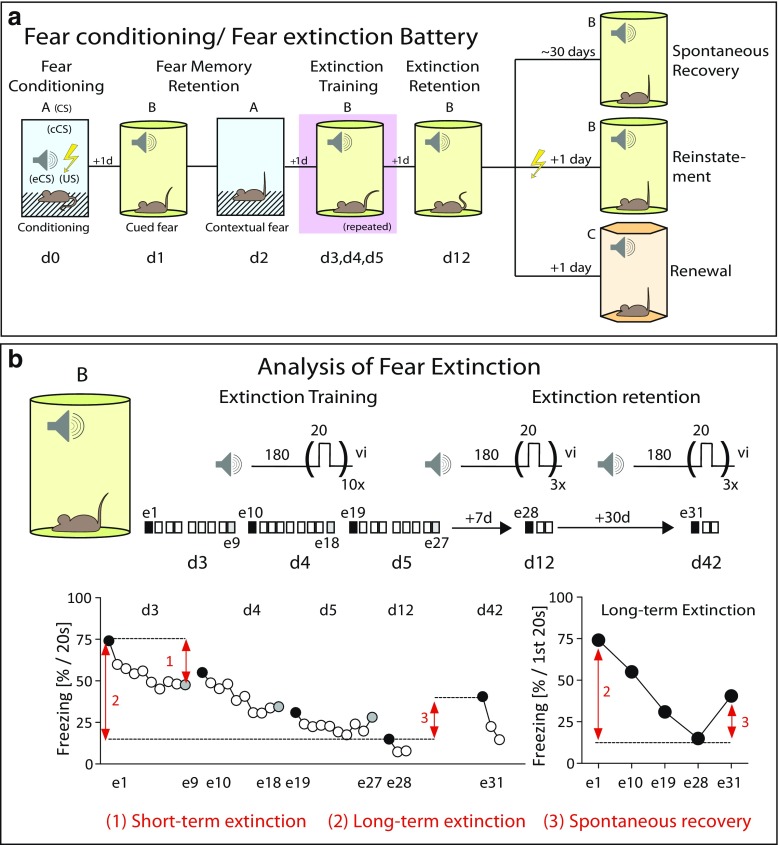
Prototypic mouse test battery for the assessment of fear extinction and recommendations for data analysis. **a** Standard conditioning protocol for the screening of phenotypes in fear conditioning (contextual and cued fear memory), fear extinction, and spontaneous recovery. After fear conditioning (d0), mice are subdued to a series of repeated testing which allow the assessment of auditory cued (d1) and contextual fear (d2), followed by repeated extinction training (d3–d5), and subsequent testing of extinction retention (d12). To demonstrate the liability of the animals to return of fear, mice either wait for another months before re-exposure to the tone (spontaneous recovery), or are re-exposed to a mild foot shock before re-exposure to the tone (reinstatement) or are re-exposed to the tone in a different context (renewal). **b** Recommendations for data analysis of fear extinction and spontaneous recovery. Extinction training is performed with multiple presentations of the tone at variable intervals on three consecutive days (e1–e27), followed by assessment of extinction retention 1 week (e28–e30) and 5 weeks later (e31–e33). (1) Short-term extinction refers to the decrease in freezing from the first to the last tone presentation on a given day. (2) Long-term extinction is measured on basis of changes in freezing to the initial tone presentation over the course of the training and retention days. (3) Spontaneous recovery describes the return of fear with the passage of time whereby changes in freezing to the initial tone presentation at d12 and d42 (e28 vs. e31) are considered (modified from Riebe et al. 2012; for further details, see text)

By definition (and experimental evidence), fear extinction is prone to relapse upon re-exposure to the conditioned stimulus in a different environment (renewal), with the passage of time (spontaneous recovery), and by re-experiencing of the unconditioned stimulus (*reinstatement*; Fig. 3a). Testing of *renewal* in a third genuine novel renewal context (ABC design, with conditioning in A, extinction training in B and renewal in C; Fig. 3a) is sometimes hampered by difficulties to design strictly different contexts. This bears the risk of false-negative findings, as it might be in case of re-testing in the original conditioning context (ABA design, with conditioning in A, extinction training in B, and renewal in A). To become aware of potential confounding influences of contextual fear, it is mandatory to report both freezing to the context right before and during tone presentation (note the importance of using the same analysis intervals). But even in this case, one has to keep in mind the complex relationship between “baseline” freezing and tone-related fear, which cannot be easily solved by simplistic arithmetic means (Jacobs et al. 2010).

There are reasons to assume that the context dependency renders extinction memories also susceptible to *spontaneous recovery* (Fig. 3a), since memories about the “safety” of the extinction context may degrade with time (Bouton 2004; Bouton et al. 2006). According to our experience, maximal extinction is achieved by multiple training sessions with variable time intervals between tone presentations during a given session and an important influence of time in between two consecutive sessions (e.g., extinction training at d1, d2, d3, and d10 after fear conditioning at d0) (Plendl and Wotjak 2010; Yen et al. 2012). Under those circumstances, I recommend to wait another month after successful completion of extinction training before assessing spontaneous recovery (e.g., d40; Fig. 3b) (Plendl and Wotjak 2010; Yen et al. 2012).

*Reinstatement* might be easily implemented but much more difficult to interpret. By definition, following successful extinction training, re-exposure to electric shock should uncover the original fear memory trace. Many studies, however, use other aversive encounters to reinstate fear memory, such as forced swim or restraint stress. It is conceivable that stressful encounters in general cause sensitization of fear responses (i.e., fear sensitization—see above) in a stimulus-unspecific manner by changing the activity level of the fear circuit. Therefore, the resulting increase in fear is of limited value for a distinction between the different processes.

All in all, challenges prone to trigger relapse of fear such as spontaneous recovery should become mandatory for every study on fear extinction. Also, one has to ascertain proper functioning of the fear circuit in particular after invasive interventions by demonstrating that the animals are still able to express fear responses. This might be achieved by re-conditioning experiments, which are preferentially either with the same (saving) or with a different conditioned stimulus, followed by testing in a different test environment.

### Data analysis in fear extinction experiments

In case of prolonged and/or repeated re-exposure to the conditioning context or conditioned stimulus, there should be clear distinctions between within-session (i.e., short-term) and between-session (i.e., long-term) extinction, and spontaneous recovery (Plendl and Wotjak 2010; Riebe et al. 2012). As illustrated (Fig. 3b), within-session extinction describes the change in fear responses over the course of the acute exposure. Between-session extinction, in turn, should be assessed by changes in *initial freezing responses* (i.e., during the first observation interval) of multiple exposure sessions (separated by ≥ 24 h). Neither freezing responses averaged over the entire session (which could be corrupted by differences in within-session extinction) (Plendl and Wotjak 2010) nor freezing responses in the end of the first exposure and in the beginning of the next exposure (which, again, could be corrupted by differences in within-session extinction which in no way relate to between-session extinction or by immobility due to resting/sleeping) should be considered. Accordingly, the spontaneous return of conditioned fear (i.e., relapse) with the passage of time (i.e., spontaneous recovery), can only refer to changes in the initial freezing responses and, thus, be indicative of liability of extinction memories (Fig. 3). I am aware that these considerations challenge current ways of data analysis and may hamper comparability of preclinical studies and experiments performed in humans. Nevertheless, I am convinced that many high-flying conclusions drawn from animal experiments would have come down to earth before they had failed in clinical studies on anxiety therapies and the prevention of return of fear.

## Sound check, stage design, and screen plot

After the discussion of multiple different processes and concerns, it is time to leave the academic corner and to enter practical grounds. To do so, I wish to suggest the following standard design for fear conditioning experiments in mice, which allow an efficient analysis of various aspects of fear memories, while keeping the number of experimental subjects at a minimum (Fig. 3a). As stated before, there is no optimal design, and the following recommendations have a subjective tone. Also, I wish to stress again the problems inherent to longitudinal studies within repeated testing of the same subjects. Key findings need replication in new cohorts of experimentally naive mice with only that key finding in focus and appropriate retention controls. As a side effect, this stepwise procedure may minimize the necessity of alpha error corrections.

### Sound check and stage design

#### Contexts

Make sure that the conditioning context and test context are sufficiently distinct. This preferentially includes differences in shape and texture of the apparatus, olfactory cues, light conditions, and structure of the floor. For instance, the floor in the test context may contain bedding which activates the animals and, thus, increases the contrast between basal immobility and freezing to the conditioned stimulus. The dimensions of the contexts should be comparable and rather small, which leads to a preponderance of freezing behavior. Do not forget to clean and olfactory “cue” the context before the first exposure (as it is done in between two tests) with a priori neutral scents.

#### Shocker

The shocker should be “calibrated” in units of individual pain thresholds. Most labs perform experiments with mutant mice. Thus, the strain used for “calibration” is preferentially the background strain of those mutants (e.g., C57BL/6N or J). The pain threshold has to be reported in addition to the shock intensity used during conditioning.

#### Speaker

The speaker has to be localized centrally above the setup. Sound pressure levels should be measured at floor levels for both conditioning and test context. I argue for sine wave tones as first choice stimulus with a frequency of 9–10 kHz and intensity between 70 and 80 dB depending on the presence of the background noise produced by the ventilator. The speakers have to properly function in a range of 6–16 kHz. Raising and falling times (e.g., 10 ms) of the control pulses will overcome the inertia of the membrane and, thus, avoid artifacts.

### The show begins

#### Fear conditioning

The number of tone–shock pairings depends on the mouse strain and shock intensity. A single tone–shock pairing may facilitate the formation of both, cued and contextual fear memory. To this end, the tone will be activated 2–3 min after insertion into the conditioning chamber and last for 20–30 s. It co-terminates with the electric shock (1–2 s; two times or three times the individual pain threshold; Fig. 1d). The temporal overlap with the tone ensures that temporal gaps between end of tone and on-set of shock are avoided, which would be characteristic for trace fear conditioning paradigms (Fig. 1c). Animals should remain in the setup for another 60 s before they are transferred back to their home cages. In this way, post-conditioning freezing can be assessed (e.g., as indicator of differences in pain perception).^3^ If using multiple setups in parallel, be aware of the risk of differences in tone and shock characteristics. To avoid that “set-up effects” are mistaken for genuine group differences, treatment groups should be equally distributed over the conditioning (and test) setups.

#### Recall of fear memories

Given the importance of sleep for memory consolidation, make sure that the animals go through a complete sleeping phase before being tested for fear memories (i.e., 1–2 days after fear conditioning). Disturbances in consolidation processes (e.g., by cage change) should be avoided within 24 h after conditioning. Memory tests begin with exposure to the test context. After a baseline period of 3 min, the tone is presented for a prolonged period of time (e.g., 3 min), without causing extinction training but enabling the assessment of short-term habituation (Kamprath and Wotjak 2004; Plendl and Wotjak 2010; Riebe et al. 2012). The next day, animals are placed back to the original conditioning/shock context in absence of any tone or foot shock, and contextual fear is assessed. Comparison of freezing to the shock context vs. the test context before tone presentation indicates the specificity of contextual fear memory (i.e., the amount of context generalization; Fig. 1e).

#### Extinction training

Another day later, extinction training can be initiated in the test context by repeated exposures (10–20) to the conditioned stimulus (same length as used during conditioning) at variable inter-tone intervals (30–120 s), starting 3 min after insertion into the context. Extinction training is repeated several times with inter-session intervals of at least 24 h. One month later, mice will be again exposed to the tone stimuli to assess spontaneous recovery of the fear memory (Fig. 3; e.g., Plendl and Wotjak 2010).

### When the music is over

#### Data analysis

If analyzed off-line from video protocols by observation, make sure that the observer is blinded to the experimental conditions. Data presentation should be based on similar observation intervals. The similarity of the observation intervals allows direct comparison of contextual and auditory-cued fear (Kim and Fanselow 1992). Analysis of freezing to the tone in time bins corresponding to the duration of the conditioned stimulus during conditioning, however, allows inferences about both the intensity of auditory-cued fear (in the beginning of tone presentation) and acute fear relief (i.e., development of the freezing response over the course of the prolonged tone presentation = short-term habituation; Fig. 1e). Changes in the initial freezing response upon extinction training serve as measure of long-term extinction and spontaneous recovery (Fig. 3b).

### Screen plot

The following screen plot can only be one of many. Of course, there can be adjustments to the individual needs. Nevertheless, I wish to summarize the basic recommendations in Fig. 3a. I fully support arguments against “over-standardization” of behavioral studies (Kafkafi et al. 2018), since this reduces the reproducibility of behavioral data. However, I would recommend to perform a few validation experiments with a given setup, before starting with the scientific project. Such experiments could include determination of the individual pain threshold of the background strain of the mutant mice under analysis. Moreover, I recommend to use the wealth of inbred strains, which are commercially available, for instance to validate hippocampus dependency of background contextual conditioning (by comparing C57BL/6 and DBA/2 mice).

All in all, I hope that the recommendations outlined in the present article will increase the comparability and reproducibility of standard fear conditioning experiments between different labs and between animal and human studies (Lonsdorf et al. 2017). With this in mind—the show can go on!

## Footnotes

1 To those willing and strong enough to envision the principles of classical conditioning on the basis of original documents including experiments in humans, I recommend to watch the movie “Mechanics of the Brain” [directed by Vsevolod Pudovkhin (1893–1959)] and produced in 1926, which can be accessed from the following Web page: https://vimeo.com/20583313.

2 It goes beyond the scope of this study to describe the neuronal basis of fear conditioning. The fuzzy term “fear circuit” stands for the multitude of brain structures implicated in generation of fear responses. This includes, among others, the amygdala complex, hypothalamus, periaqueductal gray, and medial prefrontal cortex (Silva et al. 2016; Tovote et al. 2015).

3 Many studies employ multiple tone-shock pairings and report freezing during tone presentations as evidence for intact fear memory formation, sensory and pain perception. They rarely report freezing levels right before the tone presentations. Without this information, however, the acquisition curve does not allow any inference or differentiation of tone and contextual fear.
